# Morphological and Molecular Identification of *Phytophthora capsici* Isolates with Differential Pathogenicity in *Sechium edule*

**DOI:** 10.3390/plants13121602

**Published:** 2024-06-08

**Authors:** Anell Soto-Contreras, María G. Caamal-Chan, Marco A. Ramírez-Mosqueda, Joaquín Murguía-González, Rosalía Núñez-Pastrana

**Affiliations:** 1 Facultad de Ciencias Biológicas y Agropecuarias, Universidad Veracruzana, Josefa Ortiz de Domínguez s/n, Amatlán de los Reyes 94945, Veracruz, Mexico; zS20000078@estudiantes.uv.mx (A.S.-C.); jmurguia@uv.mx (J.M.-G.); 2 CONAHCYT-Centro de Investigaciones Biológicas del Noroeste, S.C., Instituto Politécnico Nacional 195, Playa Palo de Santa Rita Sur, La Paz 23096, Baja California Sur, Mexico; mcaamal@cibnor.mx; 3 Laboratorio Agrícola-Forestal, Centro Nacional de Recursos Genéticos-INIFAP, Boulevard de la Biodiversidad 400, Rancho las Cruces, Tepatitlán de Morelos 47600, Jalisco, Mexico; marcoa.rm.07@gmail.com

**Keywords:** sporangium, ITS, *COI*, pathogenicity

## Abstract

Chayote (*Sechium edule*) is a crop of great economic and pharmaceutical importance in Mexico. Chayote is affected by *Phytophthora capsici*, which causes plant wilt and fruit rot. Three isolates of *P. capsici* (A1-C, A2-H, and A3-O) were obtained from three producing areas in Veracruz, Mexico. Morphometric characteristics of sporangia and the colony pattern on three different media were described. They were molecularly identified by amplification of the internal transcribed spacer region (ITS) and the partial sequence of cytochrome c oxidase subunit 1 (*COI*), sequences that were phylogenetically analyzed. The mating type, pathogenicity in *S. edule* fruits, and sensitivity to metalaxyl were determined. Isolate A1-C presented the largest sporangium; all sporangia were papillated, with different morphologies and pedicel lengths. All isolates showed different colony patterns: chrysanthemum (A1-C), stellate (A2-H), and petaloid (A3-O). The topology of the phylogenetic tree was similar for the ITS region and *COI* gene, the sequences of the three isolates clustered with sequences of the genus *Phytophthora* classified in group 2b, corroborating their identity as *P. capsici*. The mating type of isolates A1-C and A3-O was A2 and of isolate A2-H was A1. The pathogenicity test indicated that isolate A1-C was the most virulent and with intermediate sensitivity to metalaxyl. This work suggests that *P. capsici* isolates from various production areas in Mexico may exhibit morphological and virulence variability.

## 1. Introduction

*Phytophthora capsici* is a cosmopolitan oomycete of the *Peronosporaceae* family; it is highly destructive and dynamic, and attacks members of the *Cucurbitaceae*, *Solanaceae,* and *Fabaceae* families [1]. It has a wide genotypic diversity and an asexual cycle characterized by the production of sporangia that release unicellular biflagelated zoospores that move through moistened soils to reach their plant hosts [2]. It is a heterothallic species that has one of two mating types (A1 or A2). Both mating types are necessary for sexual reproduction [1,3], characterized by the production of oogonia and antheridia, producing oospores when fused [4].

The diversity of *P. capsici* propagules facilitates the spread of the disease, causing plant wilt, root, crown and fruit rot, and foliar blight in its hosts [3]. Under favorable environmental conditions of 25 to 30 °C and high relative humidity, the time from infection to sporulation occurs in two to three days. According to its designation as a hemibiotrophic organism, the biotrophic phase can occur during the first 24 h after infection, followed by the necrotrophic phase accompanied by the appearance of symptoms of the disease [1,3,5]. *P. capsici* produces high quantities of propagules on the surface of infected plant tissue [1], which makes the management and control of the disease difficult [6]. Furthermore, current biological methods for the management and control of the disease caused by *P. capsici* are usually ineffective, while chemical methods cause contamination of agroecosystems [7]. 

Cucurbits affected by this pathogen are cucumber (*Cucumis sativus*) [8], melon (*Cucumis melo*), squashes (*Cucurbita pepo, Cucurbita maxima, Cucurbita moschata*), watermelon (*Citrullus lanatus*) [9], and chayote (*Sechium edule*) [10]. *P. capsici* is one of the main pathogens affecting *S. edule* var. *virens levis* [11], the most commercialized varietal group and the one that is exported. 

*P. capsici* varies easily both morphologically and molecularly, favoring its spread and pathogenicity [3]. In addition, both aspects are necessary to make a correct identification, which is crucial in pathogen management to undertake control measures [12]. Among isolates of the same *Phytophthora* species, there are variations in colony morphology, which may depend on the culture medium, like V8-agar and potato dextrose agar (PDA) [13]. The genetic diversity of *P. capsici* at specific sites and across entire regions can vary dramatically [14]. For example, in the United States, the genetic diversity of *P. capsici* is likely driven by the sexual stage and production of oospores as dormant propagules reflect a high level of genotypic diversity [15]. This oomycete does not disperse through the air, creating geographically isolated subpopulations [16]. In different host plants, there are differences in the virulence of *P. capsici* isolates; this virulence can be measured by evaluating the pathogen growth rate on inoculated fruits [17,18]. Virulence can also vary depending on the genotype within the same host species [19]. 

Different *P. capsici* isolates have been characterized in Mexico. Reyes-Tena et al. (2021) evaluated the morphology of sporangia and the mating type of isolates obtained from pepper (*Capsicum annuum*), tomato (*Solanum lycopersicum*) and zucchini [19]. In the northern and central regions of Mexico, where *P. capsici* has been isolated from pepper, tomato, and cucumber, both mating types have been found, suggesting interbreeding may play an important role in the epidemiology of the disease [20]. Although *P. capsici* isolates have been obtained from chayote plants [10,11], their colony morphology, the size of asexual and sexual structures, phylogeny, virulence, and sensitivity to metalaxyl have not been characterized in detail. 

This study aimed to characterize three isolates of *P. capsici* from three main producing areas of Veracruz, Mexico, at the morphological and molecular level, phylogenetically characterizing them, determining their mating type, their virulence in fruits of *S. edule* var. *virens levis*, and their sensitivity to metalaxyl. These analyses complement each other, making the identification of this pathogen more robust, and generating information that will contribute to biotechnological and genetic management to control the disease.

## 2. Materials and Methods

### 2.1. Sampling

**Figure 1:**
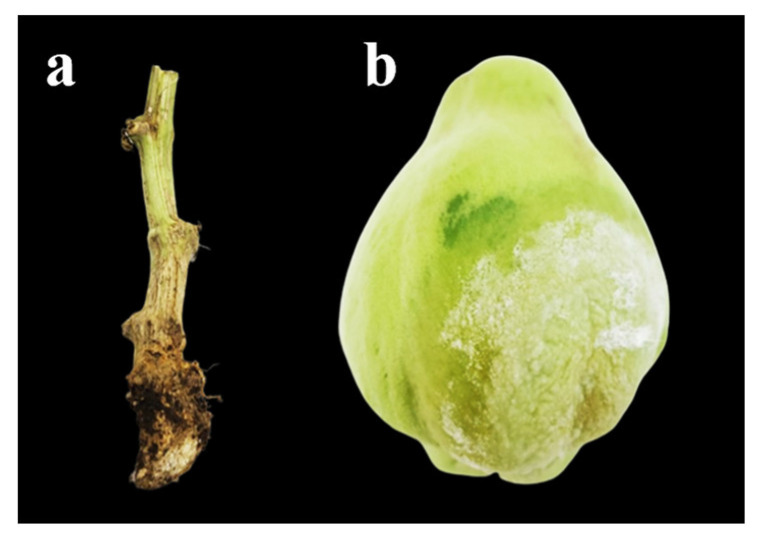
Samples of diseased *Sechium edule* tissue: (**a**) crown tissue; (**b**) fruit.

From July to September 2021, symptomatic crown fragments and fruits were collected. Symptoms included loss of turgor and the presence of mycelium in fruits; in the crown, the symptoms were necrotic lesions (Figure 1). Ten crown samples and ten fruit samples were collected from each of the studied areas (Table 1). In these areas, a high incidence of symptoms associated with *P. capsici* was reported.

### 2.2. Pathogen Isolation

The diseased tissue was washed with tap water and soap. Fragments of 5 mm^2^ from the lesion edge were cut, disinfected with 2% sodium hypochlorite for 2 min, and rinsed with sterile distilled water. Segments were cultured in Petri dishes containing water–agar and incubated at 25 °C for 2 days. A total of 15, 20, and 18 isolates were obtained from Huatusco, Coscomatepec, and Orizaba, Veracruz, Mexico, respectively. A representative morphotype was chosen from each zone for further characterization.

### 2.3. Morphological Characterization

To induce sporangia formation, the protocol by Andrade-Luna et al. (2017) was carried out with slight modifications [11]. Oomycete cultures of 7 days old in V8-agar (Campbell, Camden, NJ, USA) grown at 25 °C were used; the agar was divided into four parts, each part was placed in a sterile Petri dish, filled with sterile distilled water, and incubated under white fluorescent light at 25 °C for 5–7 days. A sample was taken and observed under a compound microscope (Nikon, Tokyo, Japan) with a micrometer. The length, width, and shape of 50 sporangia of each isolate were analyzed, and the height of their papillae was measured. Microscopic characteristics of the mycelium and its branching were also recorded. Colony morphology was determined by inoculating a 5 mm mycelial disc on V8-agar (Campbell, Camden, NJ, USA), PDA (Sigma Aldrich, St. Louis, MO, USA), and malt extract agar (MEA) (Sigma Aldrich, Steinheim, Germany), incubating in the dark at 25 °C for 7 days. Species identification was performed using a lucid key and a tabular key [4,6].

### 2.4. Molecular Identification

DNA extraction from each isolate was performed according to Johanson (1995), and 5-day-old cultures were used [21]. Two DNA regions, the ITS (internal transcribed spacer region) and the *COI* (cytochrome oxidase subunit I) gene, were amplified using the primers ITS5 (5′-GGAAGTAAAAGTCGTAACAAGG-3′) and ITS4 (5′-TCCTCCGCTTATTGATATGC-3′) and *COI* F (5′-TCAWCWMGATGGCTTTTTTCAAC-3′) and *COI* R (5′-RRHWACKTGACTDATRATACCAAA-3′) [22], respectively. PCR was performed using GoTaq^®^ G2 Flexi DNA polymerase (Promega, Madison, WI, USA) according to the manufacturer’s instructions, with 0.2 mM of each dNTP, 0.2 µM of each primer, 1.25 U of DNA polymerase, and 40 ng of DNA, with 1 mM MgCl_2_ for the ITS region and 3 mM MgCl_2_ for the *COI* gene. Amplification was performed on a MaxyGene II thermal cycler (Corning, Tewksbury, MA, USA), with the following program: initial denaturation at 95 °C for 2 min; 30 cycles at 95 °C for 1 min, 55 °C (ITS), 46 °C (*COI*) for 30 s, and 72 °C for 1 min; and 72 °C for 5 min. DNA amplifications were verified by electrophoresis in 1% agarose gels. The amplified fragments were sent for purification and sequencing in both directions to Macrogene Incorporated, Seoul, Korea. The sequences obtained were trimmed and assembled, and the consensus sequences were obtained using the BioEdit 7.2 program. They were aligned with those available in the NCBI database and deposited in GenBank.

### 2.5. Phylogenetic Analysis

Sequences for the ITS region and COI gene were selected from *Phytophthora* isolates described in Clade 2 [4,23,24]. To obtain the phylogenetic tree of the ITS region, *Nothophytophthora amphigynosa* (KY788382.1) and *N. caduca* (KY788392.1), and two species of the oomycete genus *Pythium* [*Pythium ultimum* (JN695789.1) and *P. dimorphum* (HQ643525.1)], were used as the outgroup. For the *COI* gene, the following species were considered as an outgroup: *N. amphigynosa* (KY788473.1), *N. caduca* (KY788489.1), *P. ultimum* (HQ708905.1), and *P. vanterpoolii* (HQ708993.1).

Sequence alignment was performed with the software Muscle version 7, with default parameters, and phylogenetic analysis was performed with maximum likelihood (ML) methodology together with the Kimura-2 parameter model to estimate evolutionary distances between pairs of sequences, as well as distance-based and maximum parsimony methods, inferring phylogenetic trees and 1000 bootstrap replicates for statistical support of evolutionary branch length, with the software package MEGA-7 [25].

### 2.6. Mating Type

Sexual compatibility was analyzed by culturing 7-day-old mycelial discs of each isolate and two strains with mating type A1 (CPV-259 and CPV-276) [26,27], in PDA and V8-agar. The crosses were as follows: A1-C x A2-H, A1-C x A3-O, A2-H x A3-O, A1-C x CPV-259, A2-H x CPV-259, A3-O x CPV-259, A1-C x CPV-276, A2-H x CPV-276, and A3-O x CPV-276. The mycelial discs were placed 1 cm apart, grown in triplicate on each media, and incubated at 25 °C for 21 days. The cultures were observed under a compound microscope (Nikon, Tokyo, Japan) with a micrometer. Isolates that formed antheridia, oogonia, and oospores were assigned with different mating types, and according to the known types (CPV-259 and CPV-276) were defined as A1 or A2; the diameter of 50 sexual structures was measured.

To confirm the mating type, PCRs were performed with the primers described by Li et al. (2017) [28], using DNA from the three isolates and the two reference strains in duplicate; the DNA was extracted according to the methodology described above. Final volumes of 20 µL, 40 ng of DNA, 0.2 µM of each primer, and 10 µL of super mix (Promega^®^) were used. The program used was according to the manufacturer’s recommendations: initial denaturation at 95 °C for 2 min, 30 cycles of denaturation at 95 °C for 30 s, annealing at 47.5 °C for 30 s, extension at 72 °C for 1 min, and a final extension at 72 °C for 10 min. DNA amplifications were verified by electrophoresis in 1% agarose gels. According to Li et al. (2017), mating type A1 generates an amplicon of 1121 pb and mating type A2 of 508 bp [28].

### 2.7. Pathogenicity Test

The pathogenicity test was carried out by inoculating mycelial discs from a 7-day-old colony of each isolate grown on PDA on export-quality chayote fruits at horticultural maturity. The fruits were placed in chambers of 23 cm × 19 cm × 8 cm (l × w × h), with two sterile glass jars filled with 100 mL sterile distilled water to maintain 100% relative humidity. Ten fruits per isolate were inoculated, and ten fruits with a PDA disc were used as control. The fruits remained at 25 °C for 7 days. The growth (length and width) of the lesion and the growth (length and width) of the mycelium were registered.

### 2.8. Sensitivity to Metalaxyl 

Mycelial discs of 5 mm diameter of *P. capsici* isolates A1-C, A2-H, and A3-O were grown in Petri dishes with V8-agar supplemented with 100 μg mL of metalaxyl (methyl N-(methoxyacetyl)-N-(2,6-xylyl)-DL-alaninate (Vs-Mic, Sifatec^®^, 25.10% ai). Metalaxyl was added to the previously sterilized V8-agar before pouring it into Petri dishes. The cultures were incubated in the dark at 25 °C for 5 days. The radial growth of the mycelium was evaluated every 24 h, measuring the diameter in two directions (length and width) and obtaining the average. The relative growth percentage was calculated by considering 100% of the growth of each isolate grown on V8-agar without metalaxyl. Two trials were performed with three replicates per isolate. The isolates were classified according to the scale established by Lamour and Hausbeck (2000) [29]. Sensitive (S) isolates were those with a relative growth of less than 30%, intermediate sensitivity (IS) with a relative growth of 30–90%, and insensitive (I) with a growth greater than 90%.

### 2.9. Data Analysis

In the morphological characterization, determination of mating type, and pathogenicity test, completely randomized designs were used; experiments were performed in duplicate. Data were analyzed by analysis of variance (ANOVA) followed by Tukey tests (*p* ≤ 0.05) using IBM SPSS Statistics version 25. Normality and homogeneity of variance were verified by Kolmogorov–Smirnov and Levene tests [30], respectively. When the variables did not present these parameters, they were transformed into the natural logarithm (ln).

## 3. Results

### 3.1. Pathogen Isolation and Morphological Characterization

**Figure 2:**
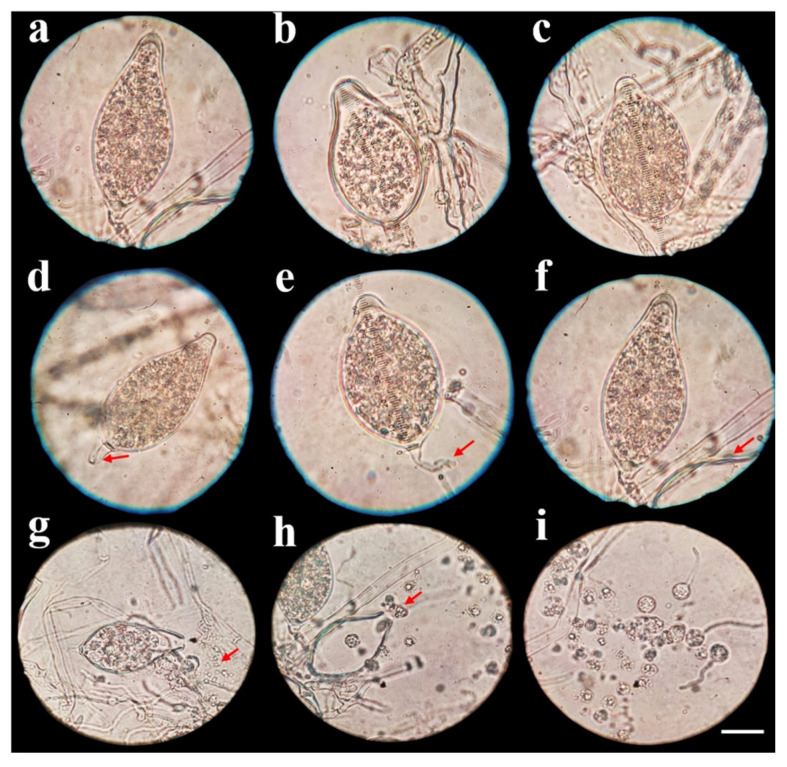
Morphology of sporangium of *Phytophthora capsici* isolates: (**a**) papillated ellipsoid; (**b**) papillated pyriform; (**c**) papillated ovoid; (**d**) papillated ovoid with short pedicel (red arrow); (**e**) papillated ovoid with medium pedicel (red arrow); (**f**) papillated ovoid with long pedicel (red arrow); (**g**) sporangium initiating zoospore release (red arrow); (**h**) sporangium finishing zoospore release (red arrow); (**i**) released zoospores, after 7 days of culture on V8-agar, bar = 10 µm.

**Figure 3:**
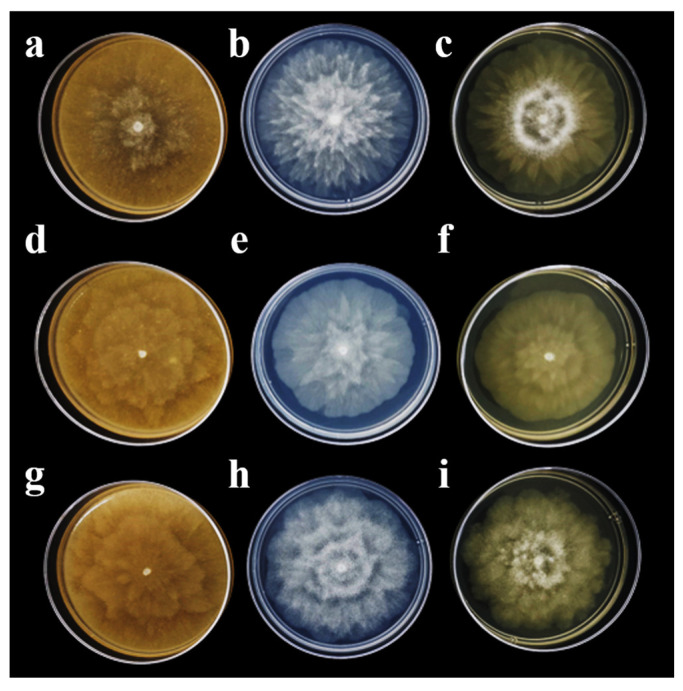
Colony morphology of *Phytophthora capsici* isolates on V8-agar, PDA, and MEA at 7 days of culture; (**a**–**c**) A1-C isolate, chrysanthemum growth; (**d**–**f**) A2-H isolate, stellate growth; (**g**–**i**) A3-O isolate, petaloid growth.

Three isolates of *P. capsici*, one from each studied area, were named A1-C = Coscomatepec, A2-H = Huatusco, and A3-O = Orizaba (Table 1). In all three isolates, the induction of sporangia was observed at 7 days of culture, and significant differences were observed in sporangia width (Table 1). However, there were no significant differences in the sporangia length and height of the papilla (Table 1). The largest sporangium size was observed in isolate A1-C with 76.43 × 43.85 (l × w) µm and a papilla height of 6.65 µm, followed by isolate A3-O with 73.91 × 42.21 µm and a papilla height of 6.38 µm, and the smallest size was observed in isolate A2-H with 73.20 × 42.01 µm and a papilla height of 6.33 µm. Three sporangium forms were observed in all isolates: papillated, ellipsoid, pyriform, and ovoid (Figure 2a–c), with short, medium, and long deciduous pedicels (5 to 80 µm) (Figure 2d–f). Moreover, sporangia releasing zoospores (10–12 µm in size) were observed (Figure 2g–i). After 7 days of growth, different colony patterns of the isolates were observed on V8-agar, PDA, and MEA. In isolate A1-C, a chrysanthemum pattern was observed, whereas in isolate A2-H, it was stellate, and in isolate A3-O, it was petaloid (Figure 3). In all three isolates, the mycelium was coenocytic, hyaline, branched, and irregular in diameter.

### 3.2. Molecular Identification

After analyzing the trimmed ITS sequence, the fragment sizes were 772 (A1-C), 648 (A2-H), and 689 pb (A3-O), and they showed 100% similarity to *P. capsici* sequences reported in GenBank (Table 2). The *COI* sequences after trimming were 646 (A1-C), 642 (A2-H), and 693 bp (A3-O); isolates A1-C and A2-H showed 100% similarity to GenBank accession numbers NC_063804.1 and MT832945.1, while the A3-O isolate showed 99.86% similarity to MN369544.1; all three reported sequences belong to *P. capsici*. The GenBank accession numbers given for ITS and *COI* sequences from this study are shown in Table 2.

### 3.3. Phylogenetic Analysis

**Figure 4:**
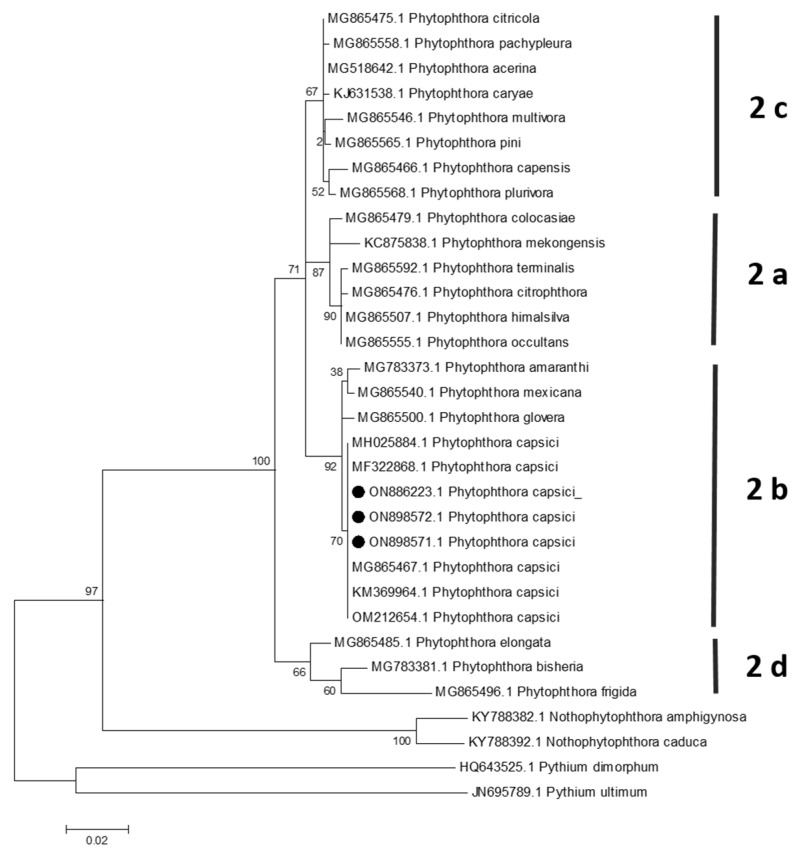
Maximum likelihood tree obtained from ITS sequences of *Phytophthora* species belonging to clade 2. The numbers on the branches indicate the support values. *N. amphigynosa*, *N. caduca*, *P. dimorphum*, and *P. ultimum* were used as outgroups. Isolates from the present study (AC-1, AH-2, and AO-3) were highlighted with a black spot. The scale bar indicates the estimated number of substitutions per site.

**Figure 5:**
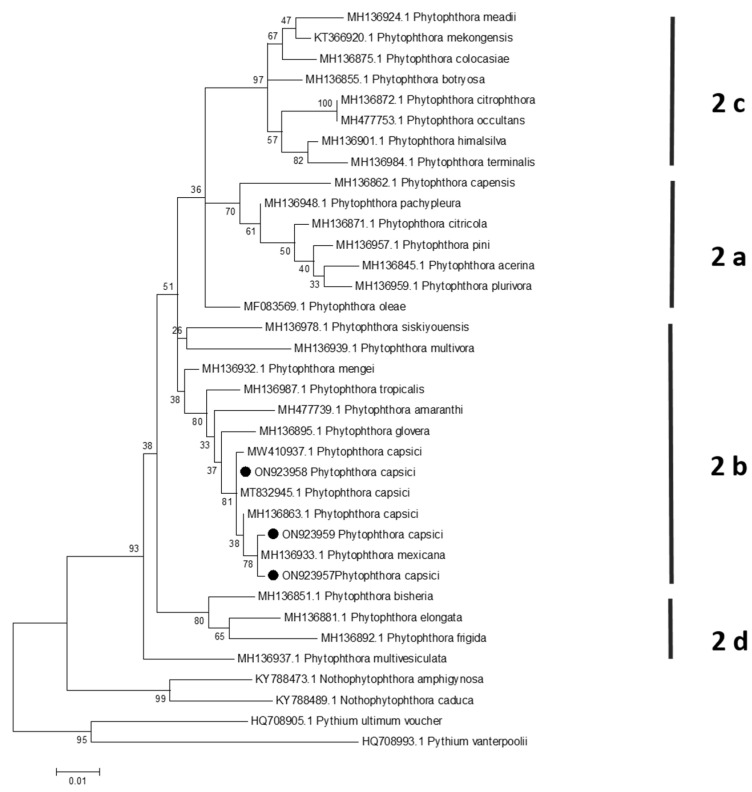
Maximum likelihood tree obtained from *COI* sequences of *Phytophthora* species belonging to clade 2. The numbers on the branches indicate the support values. *N. amphigynosa, N. caduca*, *P. dimorphum*, and *P. ultimum* were used as outgroups. Isolates from the present study (AC-1, AH-2, and AO-3) were highlighted with a black spot. The scale bar indicates the estimated number of substitutions per site.

For ITS and *COI* markers, the topology of the phylogenetic tree was similar, the sequences of the three isolates clustered with *P. capsici* sequences were classified in clade 2 (genus *Phytophthora*)/subclade 2b (*P. capsici*) (Figure 4 and Figure 5). In the case of the *COI* gene, an isolate of *P. mexicana* clustered in the same clade along with isolates of *P. capsici* (Figure 5); however, *P. mexicana* is homothallic, whereas *P. capsici* is heterothallic [4].

### 3.4. Mating Type

The mating type of isolates A1-C and A3-O was A2, and isolate A2-H was A1. In the crosses A1-C x A2-H, A2-H x A3-O, CPV-259 x A1-C, CPV-259 x A3-O, CPV-276 x A1-C and CPV-276 x A3-O, antheridia, oogonia, and oospores were produced, indicating there was sexual compatibility. Sizes of sexual structures are presented in Table 3. The diameter of oogonia ranged from 37.6 to 38.2 µm and the diameter of the oospores was 30. 9 to 31.5 µm. The largest gametangium size was obtained in the A1-C x A2-H cross. The smallest size was in the CPV-259 x A3-O cross. In the crosses A1-C x A3-O, CPV-259 x A2-H, and CPV-276 x A2-H, sexual structures were not observed. 

**Figure 6:**
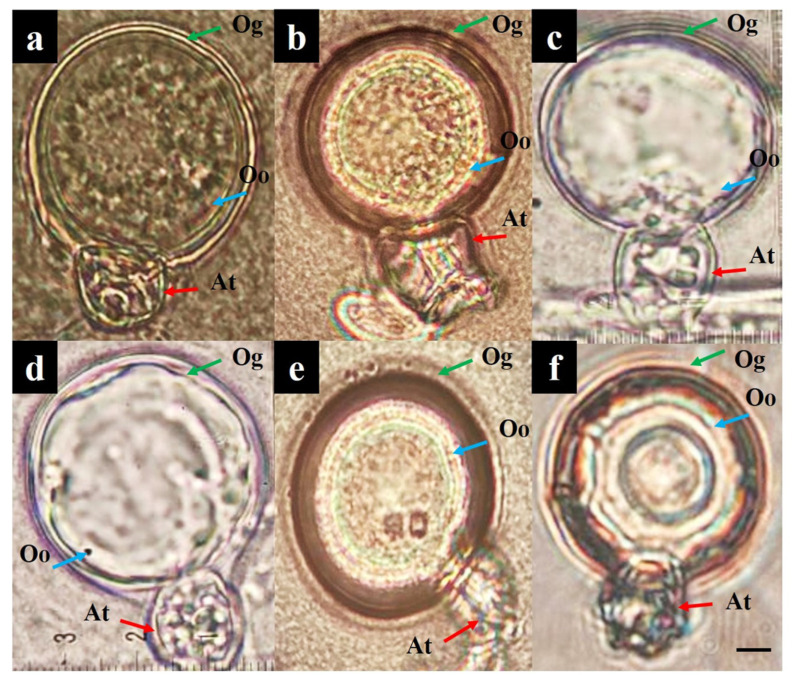
Gametangia obtained in crosses of *P. capsici* isolates: (**a**) A1-C x A2-H, (**c**) CPV-259 x A1-C, (**d**) CPV-259 x A3-O (Oo: plerotic oospore, At: amphiginous antheridium, and Og: smooth oogonium), (**b**) A2-H x A3-O, (**e**) CPV-276 x A1-C (aplerotic oospore, At: amphiginous antheridium, and Og: smooth oogonium), and (**f**) CPV-276 x A3-O (Oo: aplerotic oospore, At: amphiginous antheridium, and Og: ornamented oogonium). Bar = 10 µm.

In all crosses where there was sexual compatibility, oospores were plerotic (Figure 6a,c,d) and aplerotic (Figure 6b,e,f), antheridia were amphigenous (Figure 6a–f), and oogonia were smooth (Figure 6a–e) and ornamented (Figure 6f).

The determination of the mating type by PCR produced non-specific amplifications (Figure S1); therefore, the alignment temperature of the PCR was gradually increased (2 °C) until a single band was obtained. However, when obtaining a single band with an alignment temperature of 55.5 °C, no differences in the amplicon size were observed between the two mating types; all isolates produced a band with an approximate size of 500 bp, regardless of whether they were mating type A1 or A2 (Figure S2).

### 3.5. Pathogenicity Test

**Figure 7:**
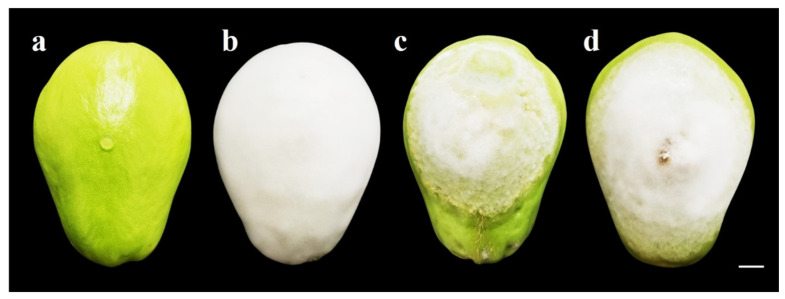
Pathogenicity of *P. capsici* isolates on *S. edule* fruits at 7 dpi: (**a**)mock inoculated; (**b**) A1-C isolate; (**c**) A2-H isolate; (**d**) A3-O isolate. Bar = 1 cm.

Symptoms began to appear 24 h after inoculation; the lesion progressed faster than the mycelium growth. At 7 days post-inoculation (dpi), severe damage (watery lesions) and the presence of the characteristic white mycelium of *P. capsici* were observed with the three isolates; however, the fruit area covered by the three isolates showed significant differences. The most pathogenic was the A1-C isolate with a lesion size of 12.81 × 9.80 cm (l × w), while the mycelium covered an area of 11.85 × 8.90 cm (Figure 7b). Isolate A3-O had intermediate pathogenicity (Figure 7d), and the least pathogenic was A2-H (Figure 7c) (Table 4). The mock-inoculated fruits remained healthy throughout the evaluation (Figure 7a).

### 3.6. Sensitivity to Metalaxyl

**Figure 8:**
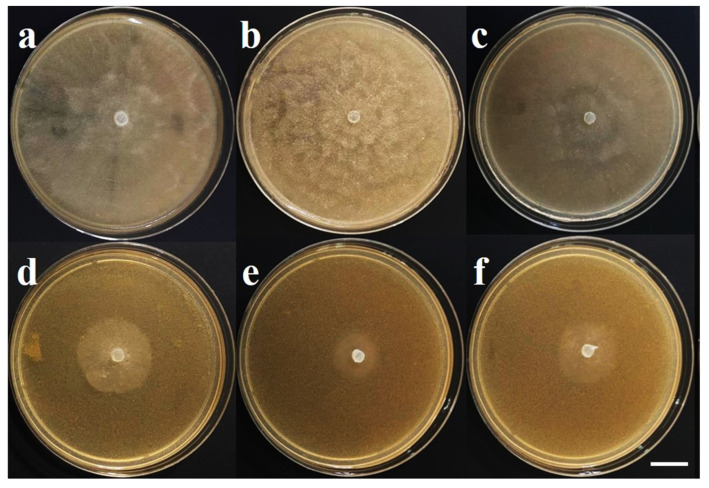
Sensitivity of *Phytophthora capsici* isolates to 100 μg mL metalaxyl after 5 days of culture; (**a**–**c**) control treatments; (**d**–**f**) cultures with metalaxyl. (**a**,**d**) A1-C isolate; (**b**,**e**) A2-H isolate; (**c**,**f**) A3-O isolate.

The sensitivity of isolates A1-C, A2-H, and A3-O to metalaxyl was observed. Isolate A1-C showed an intermediate sensitivity of 34.62%, with an average mycelial diameter of 2.85 cm on day 5 after inoculation, while isolates A2-H and A3-O of *P. capsici* showed a sensitivity of 21.98% and 25.88%, respectively (Table 5) (Figure 8). The growth pattern of the colonies of the isolates grown in the presence of metalaxyl was undefined, and malformed mycelia were observed, with short hyphae forming a rosette (Figure 8).

## 4. Discussion

In this study, *P. capsici* was isolated from three different areas in Veracruz, Mexico; the symptoms associated with the disease were plant wilting, crown rot, fruit rot with loss of turgor, and the presence of white mycelium. There are differences in the severity of the symptoms, associated with the variability of isolates or races in *P. capsici* [31]. In addition, climatological conditions for successful sample collection must be considered; this pathogen preferentially grows at 25–27 °C, with a relative humidity of 90–100% and high frequent rainfall [4]. From June to November is the ideal period for sampling and isolation of the pathogen in the state of Veracruz [10].

The first reports of *P. capsici* affecting the *Cucurbitaceae* family were observed on cucumber [8], melon, summer squash, and watermelon [32]. The isolation, identification, and management of *Phytophthora* species require the use of specialized media to generate reproductive structures [12]; in their asexual phase, the induction of sporangia and zoospores is necessary [33]. In this research, the morphological characterization of A1-C, A2-H, and A3-O isolates was achieved by the use of V8-agar, flooding, and continual light, allowing the development of sporangia and zoospores in all isolates.

The sporangium sizes in this study were larger than those reported for another *P. capsici* isolated from *S. edule* [10,11]. This variation may be due to differences in the protocols used to induce their formation, such as the culture time under continuous light; on the other hand, differences in the number of sporangia and their sizes have been found in other members of *Phytophthora* genus, which may depend on various biotic and abiotic factors [3]. Sporangia size may be directly related to pathogenicity, since zoospores are produced in these structures, which are involved in host infection. In this study, the A1-C isolate, with wider sporangia, was shown to be the most pathogenic.

In contrast to the determinations made by Reyes-Tena et al. (2020) on 41 isolates of *P. capsici* obtained from diseased pepper, tomato, and zucchini squash plants, the papilla height in the isolates obtained in this work was larger; however, the sporangia morphology was the same [26]. Aragaki and Uchida (2001) and Abad et al. (2023) also reported papillated sporangia in *P. capsici*, with ellipsoidal, pyriform, ovoid, obovoid, limoniform, globose, subglobose, fusiform, and distorted shapes, with deciduous short, medium, and long pedicels [4,34].

On the other hand, the three isolates in this work presented different colony patterns: chrysanthemum, stellate, and petaloid. González-Chavira et al. (2020) reported the same colony patterns in *P. capsici* isolated from pepper. The stellate type was the most frequent, followed by filamentous, stoloniferous, turulous, and petaloid [35]. The mycelium characteristics in this research agree with those reported in the *P. capsici* isolated from chayote [10] and pepper [35].

The morphological characterization of the three isolates was molecularly corroborated by sequencing ITS and partial sequence of the *COI* gene. These DNA regions have been widely used as a barcode for oomycete species identification [6], mainly for *P. capsici* [36], *Phytophthora citricola* [37], *Phytophthora cambivora* [38], *Phytophthora infestans* [39], *Phytophthora cinnamomi* [40], and *Phytophthora palmivora* [41]. Sixteen *Phytophthora* species in clade 1 have an identical ITS sequence, while the *COI* gene has the highest resolution of the entire genus for oomycete identification [42,43]. The amplicon sizes of ITS of this work agree with those reported in *P. capsici* isolates from chayote [10], pepper [44], and pipiana pumpkin (*Cucurbita argyrosperma* L.) [45], which had amplicon sizes from 650 to 700 pb, 580 to 796 pb, and 750 to 852 bp, respectively.

*P. capsici* is heterothallic as it has two mating types (A1 and A2); it is sterile without the interaction of different thalli of opposite mating types. To know its sexual compatibility, it is necessary to identify the presence or absence of antheridia, oogonia, and oospores in the cross of isolates. The morphology of oogonia, antheridia, and oospores, as well as their sizes, in this study agree with those reported by Abad et al. (2023) and Reyes-Tena et al. (2021) on *P. capsici* isolates [4,19].

The presence of the two mating types of *P. capsici* in a commercial orchard of *S. edule* can be devastating, since damage can increase drastically in a short time [10], making it very difficult to eradicate the pathogen. Therefore, cultural work and the application of fungicides are crucial to control *P. capsici* populations [46].

It is necessary to continue analyzing the genome of *P. capsici* to design primers that enable the identification and differentiation of the two mating types by PCR as a complementary analysis to the crosses carried out in vitro with reference strains, since the primers described by Li et al. (2017) [28] were not functional in this study.

The three isolates caused severe damage in chayote fruits at 7 dpi. However, the A1-C isolate was the most pathogenic, colonizing fruit tissue faster than the A2-H and A3-O isolates. The symptoms presented by chayote fruits were consistent with those reported on pepper fruits [17], pipiana pumpkin [44], chayote [11], tomato, and cucumber [47]. Watery lesions in fruits are developed from the hydrolytic enzyme activity of *P. capsici*, which allows it to degrade the cell wall [48]. There is evidence that there is a high correlation between enzymatic activity and pathogenicity in *P. capsici* [49].

The findings on sensitivity to metalaxyl indicated that only the A1-C isolate showed an intermediate sensitivity of 34.62%, compared to the other two isolates of *P. capsici* that were sensitive. Pons-Hernandez et al. (2020) reported 65.5% of *P. capsici* isolates from the state of Guanajuato, Mexico with intermediate sensitivity to mefenoxam and 34.5% sensitive [35]. Qi et al. (2008) reported 63.2% isolates sensitive to metalaxyl, 30.4% with intermediate sensitivity, and 6.4% resistant [50]. These results suggest that metalaxyl application can still control the damage caused by *P. capsici* in the sampled orchards. The results of the growth patterns of the colonies agree with those obtained in this study, in which the growth pattern of the colonies of the isolates with sensitive and intermediate sensitivity was indefinite, and malformed mycelia were observed, with short hyphae forming a rosette. Also, metalaxyl-sensitive isolates were the most frequent.

According to studies conducted by Lamour and Hausbeck (2001), once resistance to mefenoxam or metalaxyl has been established, the use of these should be limited, since the frequency of resistant isolates does not decrease for two years, even if the application of the chemical is eliminated and crop rotation is used [51].

## 5. Conclusions

According to morphological characterization and molecular identification, as well as phylogenetic analysis, three isolates of *P. capsici* (A1-C, A2-H, and A3-O) were obtained from three producing areas in Veracruz, Mexico, which caused fruit rot on chayote. The isolates were different in sporangium size, colony pattern, mating type, and virulence, with isolate A1-C being the most aggressive with wider sporangia and an A2 mating type. Metalaxyl can still be applied as a control of the wilt caused by *P. capsici* in chayote in the three sampled areas, since the isolates obtained showed intermediate sensitivity and sensitivity to this compound. 

The results of this study show the variability of three *P. capsici* isolates corresponding to the most predominant morphotype of each studied area; however, as future prospects, it is necessary to characterize more isolates to have a representation of the *P. capsici* population present in chayote orchards in Veracruz, Mexico, which will allow for determining the most predominant morphology, the diversity in sporangia size, as well as to know if both mating types are present in individual orchards or even in a single plant. However, these three isolates serve as valuable input to continue with future studies to obtain resistant plants to this pathogen and to study the defense mechanisms of chayote. 

## Figures and Tables

**Table 1:** Morphometric characteristics of the sporangia and papilla of 7-day-old *Phytophthora capsici* isolates grown in V8-agar flooded and under continuous light.

Isolate Code	Location	Coordinates ^1^	Sporangium Length (µm) ^2^	Sporangium Width (µm) ^2^	Papilla Length (µm) ^2^	Sporangia Form
A1-C	Tozongo, Coscomatepec, Mexico	−97.060556, 19.100556, 1660	76.43 ± 1.41 ^a^	43.85 ± 0.36 ^a^	6.65 ± 0.12 ^a^	Papillate (ellipsoid piriform ovoid)
A2-H	Tenejapa, Huatusco, Mexico	−97.004167, 19.130556, 1360	73.20 ± 1.22 ^a^	42.01 ± 0.22 ^b^	6.33 ± 0.10 ^a^	Papillate (ellipsoid piriform ovoid)
A3-O	Rincon Grande, Orizaba, Mexico	−97.078874, 18.837815, 1230	73.91 ± 1.45 ^a^	42.21 ± 0.25 ^b^	6.38 ± 0.11 ^a^	Papillate (ellipsoid piriform ovoid)

^1^ Longitude, latitude, and altitude (masl). ^2^ Values represent the mean ± standard error. Different letters in each column indicate significant differences between treatments for each variable analyzed (*p* ≤ 0.05).

**Table 2:** ITS and cytochrome c oxidase subunit I (*COI*) characterization in *Phytophthora capsici* isolates.

Isolate Code	Primers	Amplicon Size (pb)	GenBank-Accession Number of Isolates from This Study	Similarity with *P*. *capsici* Sequences in GenBank
A1-C	ITS 5/ITS 4	772	ON886223	100% (MG865467.1)
A2-H		648	ON898572	100% (MF322868.1)
A3-O		689	ON898571	100% (MH025884.1)
A1-C	*COI* F/*COI* R	646	ON923957	100% (NC_063804.1)
A2-H		642	ON923958	100% (MT832945.1)
A3-O		693	ON923959	99.86% (MN369544.1)

**Table 3:** Morphometric characteristics of *P. capsici* gametangia after 21 days of culture on PDA medium.

Crosses of Isolates and Strains	Mating Type	Oogonia Diameter (µm) ^1^	Oospore Diameter (µm) ^1^
A1-C x A2-H	A2 x A1	38.2 ± 1.5	31.5 ± 0.3
A2-H x A3-O	A1 x A2	37.9 ± 0.8	31.2 ± 1.1
CPV-259 x A1-C	A1 x A2	38.0 ± 0.5	31.1 ± 1.3
CPV-259 x A3-O	A1 x A2	37.6 ± 0.7	30.9 ± 0.4
CPV-276 x A1-C	A1 x A2	38.1 ± 1.2	31.4 ± 0.6
CPV-276 x A3-O	A1 x A2	37.7 ± 0.9	31.0 ± 1.0

^1^ Values represent the mean ± standard error.

**Table 4:** Pathogenicity of *P. capsici* isolates in chayote fruits at 7 dpi.

Isolate	Lesion Length (cm) ^1^	Lesion Width (cm) ^1^	Mycelium Length (cm) ^1^	Mycelium Width (cm) ^1^
A1-C	12.81 ± 0.07 ^a^	9.80 ± 0.06 ^a^	11.85 ± 0.06 ^a^	8.90 ± 0.03 ^a^
A2-H	8.84 ± 0.04 ^c^	5.79 ± 0.07 ^c^	7.88 ± 0.03 ^c^	4.80 ± 0.06 ^c^
A3-O	9.79 ± 0.05 ^b^	6.89 ± 0.04 ^b^	8.82 ± 0.05 ^b^	5.78 ± 0.06 ^b^

^1^ Values represent the mean ± standard error. Different letters in each column indicate significant differences between treatments for each variable analyzed (*p* ≤ 0.05).

**Table 5:** Sensitivity of *Phytophthora capsici* isolates A1-C, A2-H, and A3-O to metalaxyl after 5 days of culture.

Isolate	Length/Breadth Ratio (cm)	Sensitivity to Metalaxyl (%) ^a^	Sensitivity Scale ^a^
A1-C	2.85 ± 0.01	34.62	IS
A2-H	1.82 ± 0.01	21.98	S
A3-O	1.86 ± 0.02	25.88	S

^a^ S = sensitive, <30% of control growth; IS = intermediate, 30–90% of control growth; I = insensitive, >90% of control growth.

## Data Availability

The data reported in this paper will be available upon request.

## Supplementary Materials

The following supporting information can be downloaded at: https://www.mdpi.com/article/10.3390/plants13121602/s1.
