# Supplementary material for: Morphological and Molecular Identification of Phytophthora capsici Isolates with Differential Pathogenicity in Sechium edule
Source: Plants (Basel). 2024 Jun 8;13(12):1602. doi: 10.3390/plants13121602 (PMC11207793; doi:10.3390/plants13121602)
Supplement: Supplementary file 1 [file plants-13-01602-s001.zip › plants-2910222-supplementary.pdf]

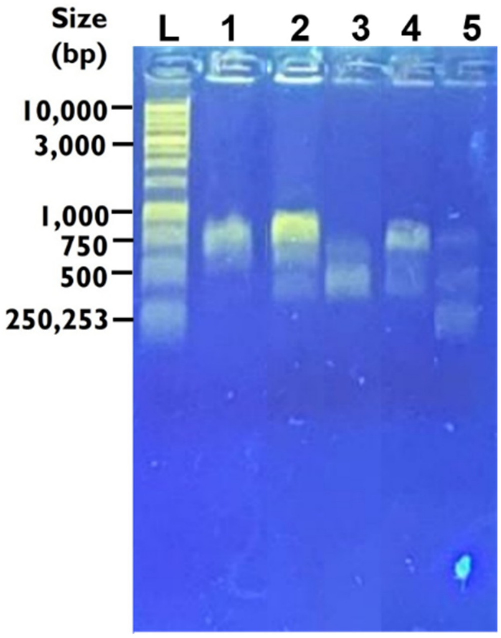

Figure S1. PCR profiles to determine the mating type of three *P. capsici* isolates using Pcap-1/Pcap-2 primers. Lane: **(L)** 1 kb ladder (Promega®, G571A); **(1)** A1-C isolate, in crosses mating type A2; **(2)** A3-O isolate, in crosses mating type A2; **(3)** A2-H isolate, in crosses mating type A1; **(4)** CPV-276 reference strain (mating type A1); **(5)** CPV-259 reference strain (mating type A1). The alignment temperature was 47.5°C.

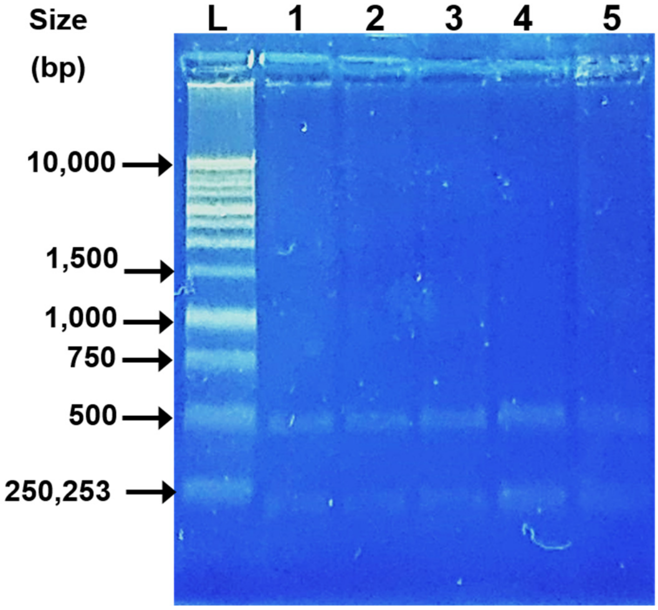

Figure S2. PCR profiles to determine the mating type of three *P. capsici* isolates using Pcap-1/Pcap-2 primers. Lane: **(L)** 1 kb ladder (Promega®, G571A); **(1)** A1-C isolate; **(2)** A3-O isolate; **(3)** A2-H isolate; **(4)** CPV-259 strain; **(5)** CPV-276 strain. The alignment temperature was 55.5°C.
